# Match and mismatch: Integrating consumptive effects of predators, prey traits, and habitat selection in colonizing aquatic insects

**DOI:** 10.1002/ece3.7181

**Published:** 2021-01-28

**Authors:** Matthew R. Pintar, William J. Resetarits

**Affiliations:** ^1^ Department of Biology Center for Water and Wetlands Resources Center for Biodiversity and Conservation Research University of Mississippi University MS USA

**Keywords:** community assembly, ecological trap, habitat selection, predation, prey vulnerability, trait‐mediated interaction

## Abstract

Predators are a particularly critical component of habitat quality, as they affect survival, morphology, behavior, population size, and community structure through both consumptive and non‐consumptive effects. Non‐consumptive effects can often exceed consumptive effects, but their relative importance is undetermined in many systems. Our objective was to determine the consumptive and non‐consumptive effects of a predaceous aquatic insect, *Notonecta irrorata*, on colonizing aquatic beetles. We tested how *N. irrorata* affected survival and habitat selection of colonizing aquatic beetles, how beetle traits contributed to their vulnerability to predation by *N. irrorata*, and how combined consumptive and non‐consumptive effects affected populations and community structure. Predation vulnerabilities ranged from 0% to 95% mortality, with size, swimming, and exoskeleton traits generating species‐specific vulnerabilities. Habitat selection ranged from predator avoidance to preferentially colonizing predator patches. Attraction of Dytiscidae to *N. irrorata* may be a natural ecological trap given similar cues produced by these taxa. Hence, species‐specific habitat selection by prey can be either predator‐avoidance responses that reduce consumptive effects, or responses that magnify predator effects. *Notonecta irrorata* had both strong consumptive and non‐consumptive effects on populations and communities, while combined effects predicted even more distinct communities and populations across patches with or without predators. Our results illustrate that an aquatic invertebrate predator can have functionally unique consumptive effects on prey, attracting and repelling prey, while prey have functionally unique responses to predators. Determining species‐specific consumptive and non‐consumptive effects is important to understand patterns of species diversity across landscapes.

## Graphical abstract

Predator–prey interactions are species‐specific; diverse prey assemblages have functionally unique responses to predator species, while predators have functionally unique effects on prey. A predatory aquatic insect disproportionately affects prey that lack effective antipredator traits that prevent predation. At the same time, some prey species avoid habitats containing predators, while other prey species preferentially colonize patches containing predators.

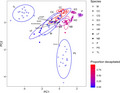

## 1 INTRODUCTION

Habitat selection, the choice of a patch based on perceived quality, is a non‐consumptive, direct effect based on individual behavior (Fretwell & Lucas, 1969). Demographic habitat selection, where habitat choices by vagile organisms are permanent, or at least long‐lived, is an especially critical non‐consumptive effect that can rival, or exceed, the impact of consumptive effects on populations, communities, and metacommunities (Abrams, 2007; Resetarits & Pintar, 2016; Resetarits et al., 2019). Predators are a particularly critical component of habitat quality, as they affect survival, morphology, behavior, population size, and community structure through both consumptive and non‐consumptive effects (Barnier et al., 2014; Creel & Christianson, 2008; Peacor & Werner, 2001; Peckarsky et al., 2008; Preisser et al., 2005; Relyea, 2001; Winnie & Creel, 2007). Thus, habitat selection in response to predation risk should decrease mortality from direct predation and reduce other predator impacts; however, benefits from predator‐avoidance responses can be offset by costs and only expressed when predation risk exceeds a threshold (Creel & Christianson, 2008; Preisser et al., 2005; Tollrian & Harvell, 1999).

For habitat selection to be effective, prey must first be able to detect, identify, and localize predators (Ferrari et al., 2010) and then accurately assess risk (Bouskila & Blumstein, 1992; Zaguri et al., 2018). Thus, we expect strong selection for those abilities, and habitat choices (preference) should closely match expected fitness (performance; Craig et al., 1989; Gripenberg et al., 2010; Rausher, 1983; Resetarits & Wilbur, 1989; Thompson, 1988). Mismatch between habitat selection preferences and fitness may result in population sinks or ecological traps, in which the cues that trigger preferences are mismatched to actual performance (Delibes et al., 2001; Hanski, 1999; Kristan, 2003; Robertson & Hutto, 2006; Schlaepfer et al., 2002).

Prey species vary in their vulnerability and behavioral responses to specific predators (Hammill et al., 2015; Klecka & Boukal, 2012; Lundkvist et al., 2003; Nilsson & Brönmark, 2000; Pyke, 1984; Resetarits & Pintar, 2016). Thus, we would expect the strength of responses to correlate with vulnerability. Specific prey traits are critical to determining vulnerability to specific predators, and the critical traits vary with predator identity, so one response does not fit all. The landscape of match and mismatch between prey responses and vulnerability has the potential to generate considerable variation in the assembly of ecological communities.

Aquatic insects are important predators in small, typically ephemeral, freshwater habitats lacking larger vertebrate predators (Wellborn et al., 1996; Wilbur, 1997). These habitats are dependent on recurrent colonization to reassemble communities after each iteration of pond drying (Merritt et al., 2008; Schneider & Frost, 1996; Wilbur, 1987). Thus, many freshwater landscapes exist as metacommunities of patches linked to each other and to the surrounding terrestrial matrix. The predominance of complex life cycles leads to networks of interactions within aquatic habitat patches, among neighboring aquatic patches, and at the terrestrial/aquatic interface. As animals colonize, they select patches based on a variety of characteristics, including resource quality and quantity (Pintar & Resetarits, 2017a, 2017b), predator presence and identity (Resetarits & Binckley, 2013; Resetarits & Pintar, 2016), competitors (Blaustein & Kotler, 1993; Pintar & Resetarits, 2020a), canopy cover (Binckley & Resetarits, 2007), and interactions and feedbacks among factors (Arav & Blaustein, 2006; Kraus & Vonesh, 2010; McNamara et al., 2021; Pintar et al., 2018). Aquatic insects, and beetles in particular, can form very diverse assemblages in small habitat patches (Jeffries, 1994; Schneider & Frost, 1996), enabling assessment of predator–prey interactions among multiple interacting species. Colonization decisions by adult aquatic beetles are critical because they can determine oviposition site and resulting offspring survival and performance, as adult dispersal can be unlikely or impossible after initial colonization due to loss of flight muscles (Johnson, 1969; Zera & Denno, 1997).

Notonectids (Hemiptera: Notonectidae) are common generalist predators in lentic freshwater habitats and play a particularly important role in fishless habitats (Cook & Streams, 1984; Wellborn et al., 1996). They are highly effective predators of a variety of aquatic taxa, including larval amphibians, zooplankton, and aquatic insects (Streams, 1987; Wilbur, 1997). While notonectids are aquatic during all life stages, adults are competent fliers and migrate among habitat patches across the terrestrial matrix (Briers & Warren, 2000). Notonectids use piercing mouthparts to feed, but due to the hard exoskeleton of adult beetles, large *Notonecta* attack beetles in a very characteristic manner, by piercing between body segments—between the thorax and abdomen or between the head and thorax—resulting in decapitation (M.R. Pintar, *personal observation*). Thus, vulnerability to predation by notonectids should depend upon the toughness of the exoskeleton and the force required to separate body segments, and/or swimming speed, either of which may correlate with size, which may also affect vulnerability directly.

We used the unique predatory signature of the most common notonectid in our system, *Notonecta irrorata*, to assess rates of predation on a diverse assemblage of aquatic beetles, and assessed how *N. irrorata* affects the colonization rate of common species and the overall beetle assemblage. We then measured the most salient phenotypic traits relating to beetle vulnerability to *N. irrorata*, and combined the variation at both the colonization stage (non‐consumptive effect) with the post‐colonization vulnerability (consumptive effect) to predict the cumulative effect of *N. irrorata* on population sizes and distribution of each species, as well as overall assemblage structure. We demonstrate that predation vulnerability is a result of a combination of highly variable beetle morphological and performance traits, while avoidance of predators via habitat selection behavior is also highly variable and not necessarily representative of vulnerability. Aquatic beetles show both adaptive, and seemingly maladaptive, responses to predation risk. Overall, *N. irrorata* has species‐specific effects on prey at both the colonization and predation stages, and these non‐consumptive and consumptive effects combine to create distinct populations and assemblages in habitat patches across a landscape.

## 2 MATERIALS AND METHODS

### 2.1 Beetle predation study

We established mesocosms (110 L plastic wading pools; 1 m diameter) on 18 May 2016 at nine sites distributed across the University of Mississippi Field Station (UMFS) with three mesocosms per site (*N* = 27) linearly arranged and separated by 1 m (edge‐to‐edge). Mesocosms contained 0.5 kg of hardwood leaf litter (primarily Fagaceae) as a resource base and were filled with unchlorinated well‐water. Mesocosms were covered with screening (1.3 × 1.13 mm opening) that was depressed below the water surface to separate colonists (above screens) from the leaf litter (below screens) and facilitate assessment of colonizing insect abundances and decapitation rates. No inocula were added from natural ponds, but zooplankton and other potential resources established rapidly as a result of immigration (Pintar & Resetarits, 2017b).

This study was naturally colonized by both predators (*N. irrorata*) and prey (beetles), thus numbers and identities of species present varied across sampling dates and mesocosms, with 0–30 *N. irrorata* colonizing each mesocosm in a given week. Colonizing beetles and *N. irrorata* assembled for seven‐day periods before we exhaustively collected all colonists, leaving the mesocosms intact. Colonists were preserved, and the majority identified to species, following (Pintar & Resetarits, 2020b). For nearly all collection dates, beetles were sorted immediately, prior to preservation, to identify any dead beetles that were not decapitated: all dead beetles had been decapitated and were recorded. All *N. irrorata* collected were live adults that had migrated from other habitats; no decapitated beetles were found in mesocosms lacking *N. irrorata*. Other colonizing hemipterans were also collected, but are excluded as we cannot assess whether *N. irrorata* were responsible for any hemipteran mortality. Sampling continued weekly until 25 July 2017 (after 14 months) when we stopped tracking decapitation rates.

As our goal was to assess decapitation rates, we only included the 122 samples that contained *N. irrorata*. We summed the number of decapitated and total beetles across these 122 samples for each species. Using these totals, we tested whether the proportion of decapitated individuals differed among the 11 most abundant beetle species (*N* > 25, to reduce potential bias of uncommon species) using an 11‐sample chi‐square test and post hoc Holm‐adjusted pairwise comparison of proportions. While this predation study lacks the rigor of a controlled predation study, the long temporal duration enabled us to capture higher abundances of some taxa, and include many more species, than we might have been able to otherwise collect at a single time for a controlled study. Therefore, while we might expect the observed mortality rates to be relatively consistent between both types of studies, we acknowledge that there could be potential issues with the lack of control and standardization in this study and present the results as illustrative of what can occur in this system. Furthermore, as the insects we study here are often found in shallow, temporary ponds (Miller & Bergsten, 2016; Wilbur, 1997), our mesocosms effectively replicate such a shallow habitat with often limited refuge or complexity. Characteristics of larger, deeper, and more complex lentic habitats may enable some taxa to use additional antipredator traits, such as diving deeper into the water and refuge use, but these habitat characteristics are not representative of our small temporary pond system or potentially characteristics that our species would have traits to take advantage of.

### 2.2 Beetle traits

To assess traits contributing to differential vulnerability to *N. irrorata*, we selected a set of traits that our previous work and observations indicated would likely be important for generating predation vulnerability. For individual beetles, we measured size (length, width), swimming ability (speed, acceleration), and the force required to separate the abdomen from the thorax (force), for each of the 11 most common species in the beetle predation study. Live adult beetles were collected at UMFS in May–June 2019 for swim trials. An arena was established using a clear plastic container (34.6 × 21.0 × 12.4 cm) filled with 0.5 L of well‐water held at room temperature (24.8°C), and placed above a light pad to provide backlight and contrast, with a scale for calibration. Individual beetles were placed into the middle of the arena and allowed to swim while a Nikon D3300 (above the arena) recorded two 15 s videos at 60 fps. Videos were analyzed (frame‐by‐frame) using Tracker v 5.0.7 to record speed and acceleration (Brown, 2019). For each beetle, we determined the maximum average speed recorded across ten continuous frames and the average of the 20 highest acceleration values. Although this arena is far less complex than most natural ponds, it enables consistent and standardized assessment of swimming abilities in a novel and somewhat stressful environment, which enabled us to capture higher swimming speeds and accelerations, as would be expected in the presence of a predator. Furthermore, while behavior of individuals can vary greatly across the trial period, we only assess averages of maximum speeds and accelerations for each individual to determine peak abilities.

After swim trials, beetles were euthanized in 70% ethanol and photographed using a Dino‐Lite for determination of maximum length and width using ImageJ (Schneider et al., 2012). We then used an M&A Instruments HF‐50 Digital Push‐Pull Force Gauge (50 N capacity, accuracy 0.01 N) with a chisel tip to determine the minimum force required to separate the abdomen from the thorax. Beetles were placed horizontally on a flat surface and held in place as the chisel tip was pressed into the beetle between the elytra (abdomen) and pronotum (thorax), perpendicular to the beetle surface at the point. This process mimics the form of attack we have observed *N. irrorata* use when preying on beetles, resulting in decapitation. Beetle identifications were verified after all tests were complete. The force required for two species, *Enochrus ochraceus* and *Paracymus* (both Hydrophilidae), was below the force gauge's 0.50 N response threshold and was estimated using the linear relationship between force and length that was consistent across two other hydrophilids, *Cymbiodyta chamberlaini* and *Tropisternus lateralis*. We aimed to test 15 individuals of each species (9 species had 14–16 individuals), but *Neoporus blanchardi* had only 9 individuals. During the testing period, we found only one *Hydrocolus oblitus*, so we included 8 individuals of this species collected in April 2019 (preserved in 70% ethanol) and estimated the swimming speed and acceleration using the linear relationship between these two variables and length from the one individual we did test. We also assessed the swimming ability and size of adult *N. irrorata* for comparison.

We assessed differences in each of the log‐transformed traits among species using mixed effects models with species as a fixed effect and test date as a random effect, with post hoc Holm‐adjusted Tukey comparisons. Variation in the five traits among species was visualized using PCA. We used logistic regression to assess the effects of traits on the observed decapitation rates. However, because of the number of traits measured (5), the number of species (11), and interactions, we used factor analysis to generate three aggregate variables used in logistic regression, one for each category of variable: size, swimming, and force. We lacked sufficient degrees of freedom to work with all five traits and interactions in our logistic regression, as each of the eleven species contributes one degree of freedom. Therefore, we first log‐transformed all individual traits and scaled them with the “scale” function in R. We then used these five scaled trait variables in a factor analysis (“fa” function, psych package) fit with factoring method of “uls,” rotation set to “varimax,” and three output factors. Using the standardized loadings output from the factor analysis (Table 1), we excluded loadings < 0.6 and used the remaining loadings to adjust the original scaled variable values and combined loadings for each ULS factor to generate aggregate variables using the formulas in Table 1. These three aggregate variables and all possible interactions were used as predictor variables in logistic regression.

**TABLE 1:** Standardized loadings from the factor analysis on scaled beetle traits. Loadings < 0.6 were excluded, and the remaining loadings (bold) were used to adjust the original scaled variable values to generate three aggregate variables using the formulas listed below

Trait	ULS1	ULS2	ULS3
Force	0.02	0.23	**0.97**
Speed	0.35	**0.89**	0.29
Acceleration	0.53	**0.71**	0.19
Length	**0.92**	0.36	−0.01
Width	**0.95**	0.31	0.06

Note

ULS1 = Size = Length × 0.92 + Width × 0.95.

ULS2 = Swim = Acceleration × 0.71 + Speed × 0.89.

ULS3 = Force = Force × 0.97.

### 2.3 Habitat selection experiment

Our habitat selection experiment was conducted during peak abundance of dispersing beetles. Adult *Notonecta irrorata* were collected on 23 May 2017 from one fishless pond at UMFS (34°25′09.13″ N, 89°23′37.76″ W). On 24 May we established mesocosms as in the beetle predation study, but with 70 L pools linearly arranged (0.85 m diameter), each containing 0.25 kg of hardwood leaf litter. To reduce the probability of notonectid colonization, mesocosms were established at three sites (blocks) where notonectids rarely appeared in prior experiments. Treatments consisted of three densities of *N. irrorata*: 0, 2, or 10 individuals per mesocosm with nine replicates per treatment, three per block (*N* = 27). The *N. irrorata* densities represent low and high densities commonly encountered in natural ponds and mesocosms at UMFS, although *N. irrorata* can be absent or occur at higher densities. Treatments were randomly assigned to the first mesocosm in each block, with treatments in the second mesocosm also randomly assigned from the two other treatments. The third mesocosm was assigned the only remaining treatment, and then the remaining mesocosms in a block were assigned treatments in the same order as the first three.

*Notonecta irrorata* were randomly assigned and added to mesocosms on 24 May and placed below the screens to prevent them from consuming any colonists. The water surface was accessible to *N. irrorata* along the sides of the mesocosms, but they were unable to escape because screens were tightly fit to the top rim and exterior sides of mesocosms. No inocula were added, and mesocosms were immediately opened for colonization after *N. irrorata* addition. Mesocosms were checked daily for colonizing notonectids, which were immediately removed (only two colonized). Beetles were exhaustively collected weekly, preserved, and identified as in the beetle predation study.

Because dytiscids may selectively colonize habitats with more zooplankton (Pintar & Resetarits, 2017b), which are potential prey, we measured water chemistry, sampled zooplankton, and terminated the experiment on 28 June. Water chemistry of each mesocosm was measured prior to final sampling (to avoid disturbance) with a YSI Professional Plus meter. For zooplankton, we collected two 400 ml water samples from separate locations in each mesocosm, filtered through 80 μm mesh into 50 ml centrifuge tubes, and preserved with Lugol's solution. We counted zooplankton within 1 ml subsamples from each 50 ml sample (Wetzel & Likens, 2000). The experiment was terminated on 28 June, when we searched through the leaf litter to determine *N. irrorata* survival, which was 100%.

We analyzed the cumulative number of beetles that colonized the experiment, and that of the six most abundant species (*N* > 45). Not all beetle species from the beetle predation study were present in this experiment due to typically strong temporal and spatial variation in beetle abundances. We assessed beetle species richness with abundance as a covariate. For all nine taxa analyzed, we set a priori contrasts to first compare mesocosms with no *N. irrorata* to those that contained *N. irrorata* (both 2 and 10 per mesocosm), and second to compare 2 *N. irrorata* and 10 *N. irrorata* treatments. We used mixed effects models fit by maximum likelihood with treatment as a fixed effect and block as a random effect. Zooplankton abundances were initially included as a covariate, but had no significant effects (*p* > 0.30) and were excluded. Individual species are largely expected to be independent as colonizing adult beetles do not typically respond to the presence of other beetle within patches (Pintar & Resetarits, 2020a). We analyzed water chemistry similar to other variables with mixed effects models fit by maximum likelihood, treatment as a fixed effect, block as a random effect, and contrasts that first tested between mesocosms with *N. irrorata* and those without, then between mesocosms with 2 *N. irrorata* and those with 10 *N. irrorata*. Conductivity, dissolved oxygen, and temperature were log‐transformed, while pH was not transformed. The dissolved oxygen analysis included temperature as a covariate. We analyzed community structure (Bray‐Curtis distances) of the entire beetle assemblage and of the six most common species with PERMANOVA, and beta diversity with PERMDISP, with NMDS for visualization. We then simulated what these assemblages would look like by adjusting the colonization totals by the proportion that were decapitated in the predation study (Table 2).

**TABLE 2:** We adjusted the beetle assemblages from the colonization experiment by the decapitation rates observed in the beetle predation study to project what these assemblages would like incorporating both habitat selection and predation. For structurally similar species, we adjusted the colonization rates by the decapitation rate of common, similar species. If a species from the colonization experiment had no structurally similar species in the beetle predation study (NA), it remained unadjusted (adjustment rate = 1)

Colonizing species	Species used to determine adjustment rate	Adjustment rate (rounded)
Dytiscidae		
*Celina hubbelli*	NA	1
*Copelatus chevrolati*	*Copelatus chevrolati*	1
*Copelatus glyphicus*	*Copelatus glyphicus*	0.0463
*Desmopachria*	NA	1
*Hydaticus bimarginatus*	*Hydaticus bimarginatus*	1
*Hydroporus rufilabris*	*Hydroporus rufilabris*	0.6667
*Laccophilus fasciatus*	*Laccophilus fasciatus*	0.985
*Laccophilus proximus*	*Laccophilus proximus*	1
*Neoporus blanchardi*	*Neoporus blanchardi*	0.4429
*Uvarus lacustris*	*Uvarus lacustris*	1
Haliplidae		
*Peltodytes sexmaculatus*	*Peltodytes sexmaculatus*	1
Hydraenidae		
*Hydraena marginicollis*	NA	1
Hydrophilidae		
*Berosus exiguus*	NA	1
*Berosus infuscatus*	*Berosus infuscatus*	1
*Cymbiodyta bifidus*	*Cymbiodyta chamberlaini*	0.0725
*Cymbiodyta chamberlaini*	*Cymbiodyta chamberlaini*	0.0725
*Cymbiodyta vindicata*	*Cymbiodyta chamberlaini*	0.0725
*Enochrus hamiltoni*	*Enochrus ochraceus*	0.6018
*Enochrus ochraceus*	*Enochrus ochraceus*	0.6018
*Enochrus perplexus*	*Enochrus ochraceus*	0.6018
*Enochrus pygmaeus*	*Enochrus ochraceus*	0.6018
*Helochares maculicollis*	*Cymbiodyta chamberlaini*	0.0725
*Hydrochara soror*	NA	1
*Paracymus*	*Paracymus*	0.7844
*Tropisternus collaris*	*Tropisternus collaris*	1
*Tropisternus lateralis*	*Tropisternus lateralis*	1

### 2.4 Data analysis software

All univariate analyses were conducted in R v 4.0.3 (R Core Team, 2020) using the lme4 v 1.1‐26 and lmerTest v 3.1‐3 packages for mixed effects models (Bates et al., 2015; Kuznetsova et al., 2017), with the multcomp package v 1.4‐15 (Hothorn et al., 2008) for post hoc comparisons and the psych package v 2.0.12 (Revelle, 2020) for factor analysis. PERMANOVA, PERMDISP, and NMDS were performed with Primer 7 and the PERMANOVA+ add‐on (Anderson et al., 2015; Clarke & Gorley, 2015).

## 3 RESULTS

### 3.1 Beetle predation study

**FIGURE 1:**
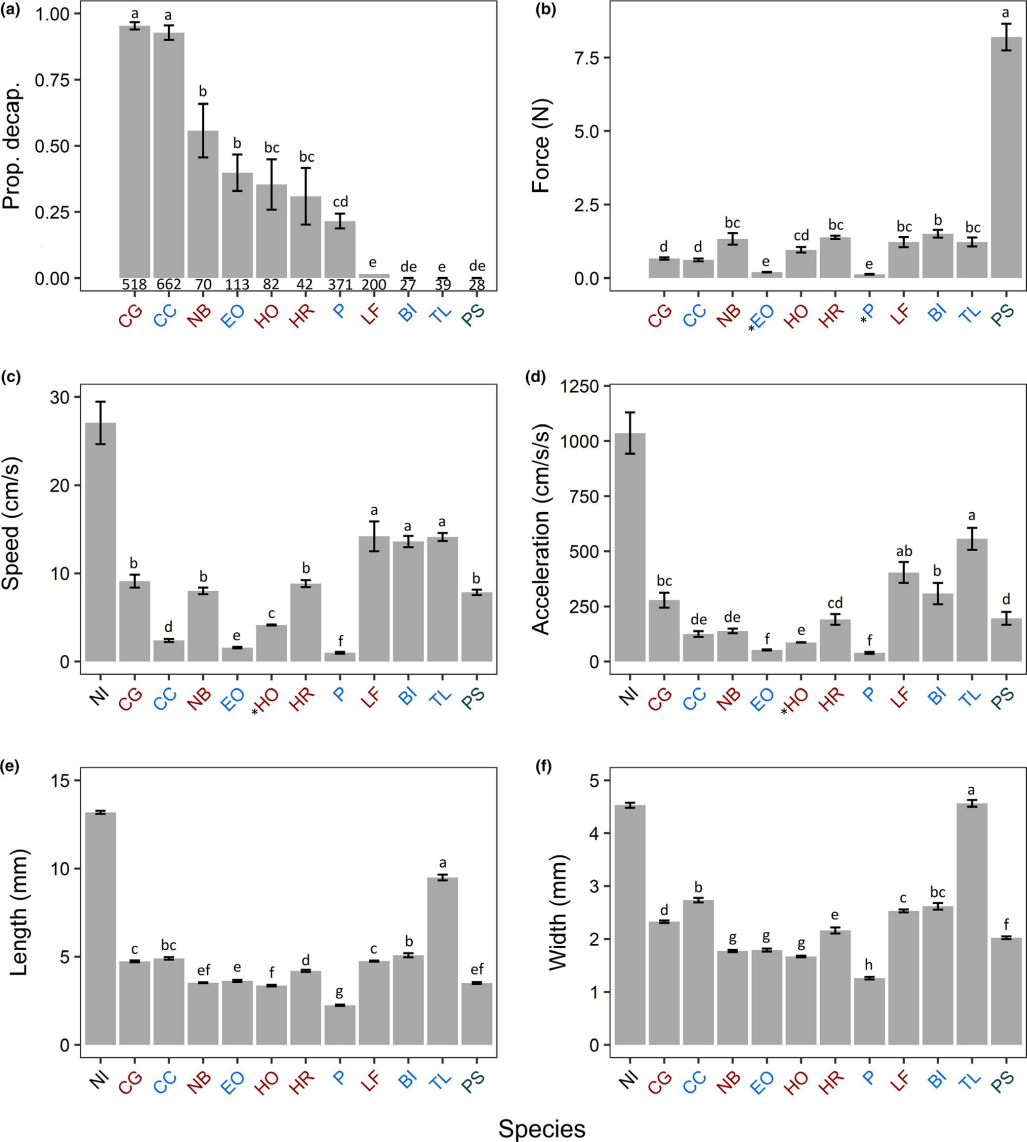
(a) Proportion of individuals of the eleven most abundant beetle species (*N* > 25) found decapitated in patches with *N. irrorata*. Numbers below the bars indicate the total number of beetles of each species that colonized these patches (see Table 3). (b–f) Data from beetle trait analyses showing average (±*SE*) (b) index of resistance to decapitation (force required to separate the pronotum and thorax), (c) peak swimming speed, (d) peak swimming acceleration, (e) length, and (f) width. Species names colored by family (blue = Hydrophilidae; red = Dytiscidae; green = Haliplidae; see also Table 3). Species are arranged from left to right in decreasing order of decapitation rates. *Notonecta irrorata* included in (c–f) for comparison, but not included in analyses. Error bars on (a) are standard error estimates based on average decapitation rates per sample, while the bars themselves are the cumulative proportions decapitated, not an average per sample. Letters over bars indicate statistical groupings. BI = *Berosus infuscatus*, CC = *Cymbiodyta chamberlaini*, CG = *Copelatus glyphicus*, EO = *Enochrus ochraceus*, HO = *Hydrocolus oblitus*, HR = *Hydroporus rufilabris*, LF = *Laccophilus fasciatus*, NB = *Neoporus blanchardi*, NI = *N. irrorata*, P = *Paracymus*, PS = *Peltodytes sexmaculatus*, TL = *Tropisternus lateralis*

The beetle predation study was colonized by 12,482 beetles of 67 species; 337 *N. irrorata* were collected from 122 samples (1 sample = all insects from one mesocosm/week). Zero beetles were decapitated in the 1,498 samples without *N. irrorata*, establishing *N. irrorata* as responsible for observed decapitation of aquatic beetles. We limited our dataset to the samples containing *N. irrorata* (*N* = 122), which contained 2,341 beetles of 39 species in six families (Table 3). Twelve species had at least one decapitated individual, while 27 had no decapitations. Among the eleven species (in three families) above our analysis threshold (*N* > 25), there was significant variation in the proportion decapitated (χ^2^ = 1,286, *df* = 10, *p* < 0.0001). Three species had no decapitated individuals: two hydrophilids, *Berosus infuscatus* (BI) and *Tropisternus lateralis* (TL), and one haliplid, *Peltodytes sexmaculatus* (PS; Figure 1a). *Laccophilus fasciatus* (LF; Dytiscidae) had nearly no decapitation (3/200 individuals). The two most abundant species, the hydrophilid *Cymbiodyta chamberlaini* (CC) and the dytiscid *Copelatus glyphicus* (CG), had the highest decapitation rates (92.7% and 95.4%, respectively). Five other species (hydrophilids and dytiscids) had intermediate rates ranging from 21.6% (*Paracymus*; P) to 55.7% (*Neoporus blanchardi*; NB).

**TABLE 3:** List of beetle species that colonized *Notonecta irrorata*‐containing mesocosms in the beetle predation study (excludes all samples without *N. irrorata*). Decap indicates the number of individuals of that species that were decapitated. Total indicates the total number of individuals collected (including decapitated)

Taxa	Decap	Total	Taxa	Decap	Total
**Dytiscidae**			**Haliplidae**		
*Acilius fraternus*	0	1	*Peltodytes muticus*	0	2
*Acilius mediatus*	0	14	*Peltodytes sexmaculatus*	0	28
*Bidessonotus inconspicuus*	0	1	**Helophoridae**		
*Copelatus chevrolati*	0	15	*Helophorus linearis*	0	3
*Copelatus glyphicus*	494	518	**Hydraenidae**		
*Coptotomus loticus*	0	18	*Hydraena marginicollis*	0	10
*Hydaticus bimarginatus*	0	15	**Hydrochidae**		
*Hydrocolus deflatus*	1	14	*Hydrochus rugosus*	0	1
*Hydrocolus oblitus*	29	82	**Hydrophilidae**		
*Hydroporus brevicornis*	7	8	*Berosus infuscatus*	0	27
*Hydroporus rufilabris*	14	42	*Berosus sayi*	0	4
*Ilybius biguttulus*	0	7	*Cymbiodyta chamberlaini*	614	662
*Laccophilus fasciatus*	3	200	*Cymbiodyta vindicata*	0	1
*Laccophilus proximus*	0	9	*Enochrus consortus*	1	6
*Meridiorhantus calidus*	0	5	*Enochrus fimbriatus*	1	4
*Neoporus blanchardi*	39	70	*Enochrus ochraceus*	45	113
*Platambus flavovittatus*	0	13	*Enochrus pygmaeus*	0	1
*Thermonectus basillaris*	0	9	*Helochares maculicollis*	0	2
*Uvarus granarius*	0	2	*Hydrochara soror*	0	4
*Uvarus lacustris*	0	4	*Paracymus*	80	371
			*Tropisternus blatchleyi*	0	5
			*Tropisternus collaris*	0	11
			*Tropisternus lateralis*	0	39

### 3.2 Beetle traits

**FIGURE 2:**
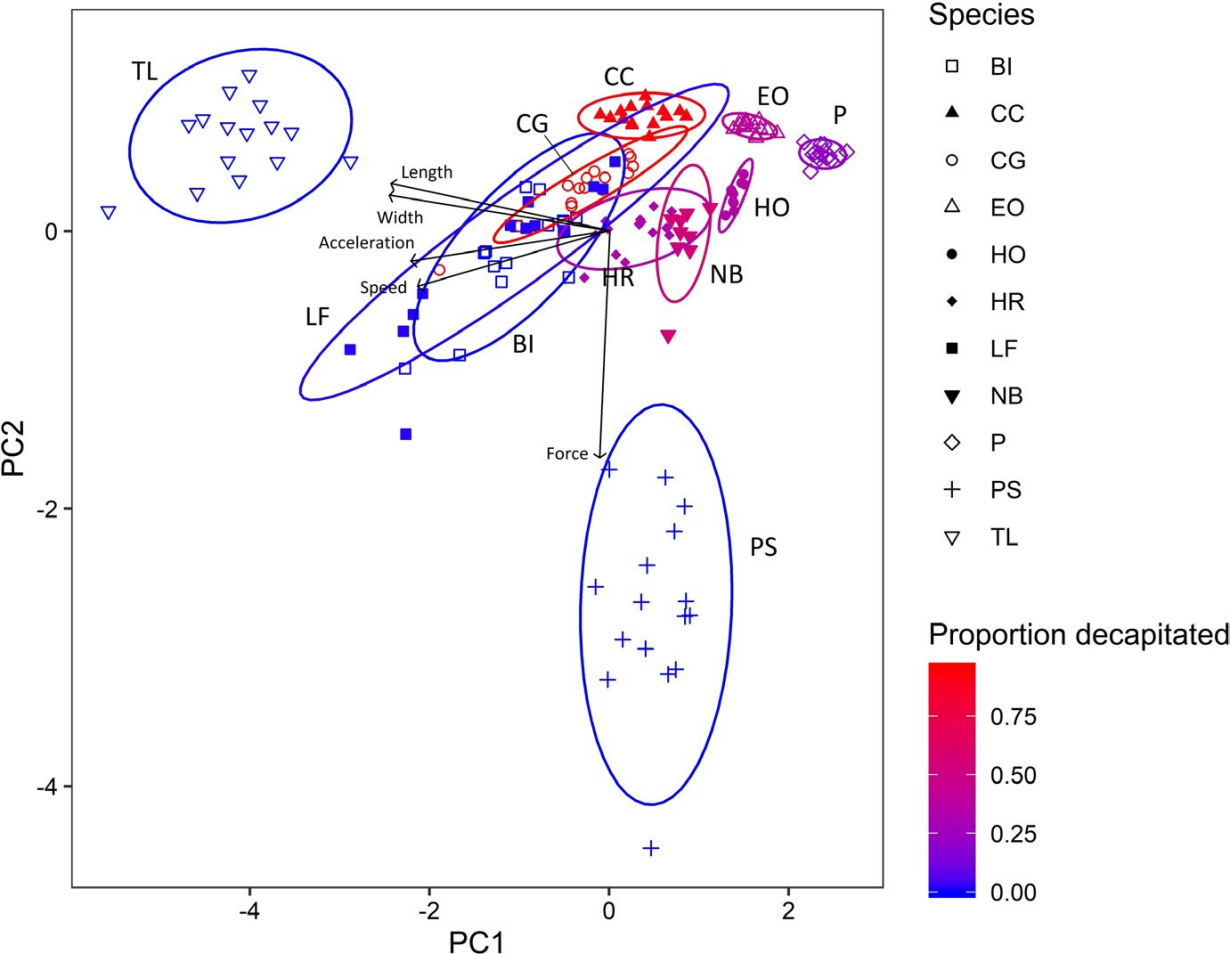
Principal components analysis of the five beetle traits: force, speed, acceleration, length, and width. Symbol shapes represent species (also labeled), and color is gradient of decapitation rates (Figure 1a). Ellipses are 95% confidence ellipses for each species based on multivariate normal distribution. PC1 accounts for 62.85% of the variance and PC2 21.63% (see Table 5)

There was significant variation in all five traits among beetle species, both within and among families (*p* < 0.0001 for all traits; Table 4). Swimming speed was slowest for three hydrophilids, with three dytiscids and *P. sexmaculatus* having intermediate speeds, while two hydrophilids and *L. fasciatus* (LF) had the fastest swimming speeds, though still about half the *N. irrorata* average (Figure 1c). Acceleration largely paralleled speed, with the highest acceleration rates still half that of *N. irrorata* (NI; Figure 1d). Maximum length and width (Figure 1e,f) illustrate how most of the common beetle species are much smaller than *N. irrorata*, with the exception of *T. lateralis* (TL). The force required to separate body parts for eight of the eleven species ranged from 0.5 to 2 N, with two hydrophilids requiring less than 0.5 N and *P*. *sexmaculatus* (PS) over 7.5 N (Figure 1b). The PCA (Figure 2) illustrates how the five measured traits cumulatively affected the decapitation rate for each species. Speed, acceleration, length, and width were all important components of PC1 (all loadings ~−0.5; Table 5), while force was the dominant component of PC2 (loading = −0.9), collectively accounting for 84.5% of the variance. This results in considerable spatial separation of the largest (*T. lateralis*) and most resistant species (*P. sexmaculatus*), with further separation based on speed (*B. infuscatus*, *L. fasciatus*, *T. lateralis*), and smaller sizes (*E. ochraceus*, *Paracymus*, *H. oblitus*). Logistic regression indicates that these traits and their interactions have a clear, strong role in generating the observed decapitations rates of beetles (Table 5c).

**TABLE 4:** Results of the mixed effects analyses of the five beetle traits. Bold indicates statistical significance (*p* < 0.05)

Analysis	SS	Num *df*	Den *df*	*F*	*p*	*R* ^2^
Force	41.558	10	90.0	183.36	**<0.0001**	0.9335
Speed	75.668	10	89.8	201.42	**<0.0001**	0.9335
Acceleration	97.285	10	64.4	64.314	**<0.0001**	0.8116
Length	12.77	10	155	627.75	**<0.0001**	0.9759
Width	8.0316	10	155	453.76	**<0.0001**	0.9670

Note

*R* ^2^ here is the correlation between the fitted and observed values.

**TABLE 5:** (a) Importance of components in the PCA of the five beetle trait variables. (b) Loadings for the five trait variables across the five components in the PCA. (c) Results of logistic regression for three aggregate trait variables. Bold indicates statistical significance (*p* < 0.05)

(a)	PC1	PC2	PC3	PC4	PC5
Standard deviation	1.7727	1.0400	0.7414	0.45753	0.13058
Proportion of variance	0.6285	0.2163	0.1100	0.04187	0.00341
Cumulative proportion	0.6285	0.8448	0.9547	0.99659	1.0000

(b)	PC1	PC2	PC3	PC4	PC5
Acceleration	−0.4961	−0.1105	−0.4132	−0.7556	−0.0014
Speed	−0.4629	−0.2594	−0.5513	0.6435	−0.0203
Force	−0.0220	−0.9212	0.3810	−0.0593	0.0464
Length	−0.5198	0.2123	0.4095	0.0849	0.7141
Width	−0.5189	0.1636	0.4610	0.0658	−0.6982

(c)	Estimate	*z*	*p*		
Size	−0.3803	−1.444	0.1486		
Swim	−0.5158	−2.419	**0.0156**		
Force	−5.7629	−10.516	**<0.0001**		
Size:Swim	−2.7909	−4.133	**<0.0001**		
Size:Force	2.5391	0.954	0.3399		
Swim:Force	−3.8453	−5.322	**<0.0001**		
Size:Swim:Force	−1.6582	−5.673	**<0.0001**		

### 3.3 Habitat selection experiment

**FIGURE 3:**
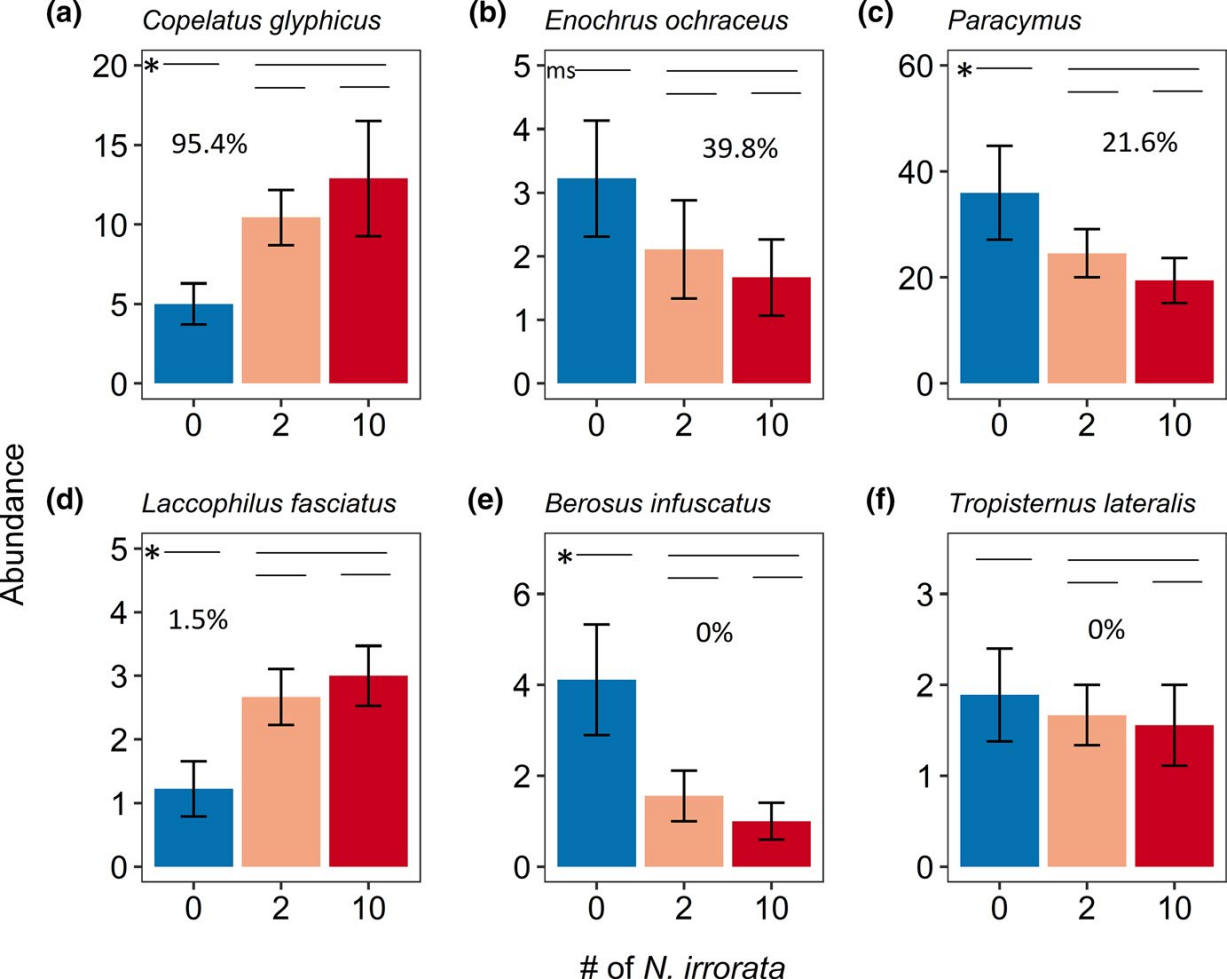
Average total (±*SE*) colonists per mesocosm of the six most abundant beetle species (*N* > 45) in the colonization experiment across three densities of *N. irrorata*. Lines above the figures illustrate the a priori contrasts: top line contrasts control mesocosms with those containing *N. irrorata* (both 2 and 10 individuals), and bottom line contrasts mesocosms with 2 versus 10 *N. irrorata*. Asterisks indicate significant contrasts (*p* < 0.05); ms indicates marginal significance (0.05 < *p* < 0.1). Percentages indicate the percent of individuals decapitated in the predation study (see Table 3, Figure 1)

**FIGURE 4:**
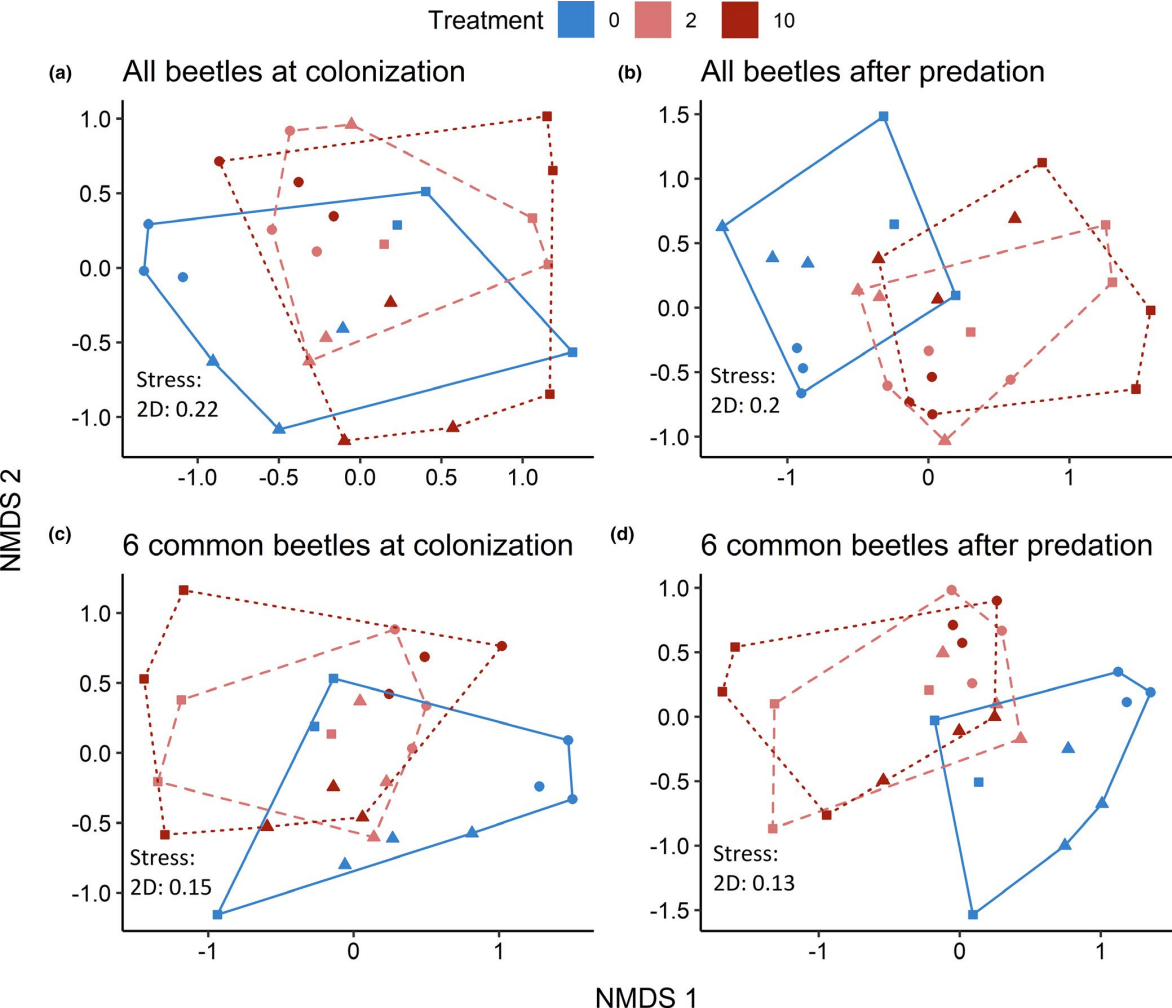
Nonmetric multidimensional scaling of (a) the entire beetle assemblage in the colonization experiment. (b) the predicated change to the entire beetle assemblage following predation by *N. irrorata*, calculated with decapitation rates from the predation study (Figure 1a, Table 2). (c) is a NMDS of the six most common beetle species in the colonization experiment, while (d) is a NMDS of those six species adjusted with decapitation rates from the predation study. Line type and color represent the three treatments from the colonization experiment (0, 2, 10 *N. irrorata* per mesocosm), while shape represents blocks, illustrating spatial variation in assemblage structure. Kruskal fit scheme = 1 for all figures

A total of 1,305 beetles of 26 species in four families colonized the experiment (Table 6), with six species abundant enough for analysis. Two hydrophilids, *B. infuscatus* and *Paracymus*, colonized mesocosms without *N. irrorata* at higher rates than those with *N. irrorata*, while *E. ochraceus* was marginally higher (Table 6; Figure 3). Two dytiscids, *C. glyphicus* and *L. fasciatus*, colonized mesocosms with *N. irrorata* at higher rates than those without *N. irrorata*. One hydrophilid, *T. lateralis*, did not exhibit any colonization differences. No species showed differences between 2 and 10 *N. irrorata* treatments, indicating threshold responses at relatively low *N. irrorata* density. There were no differences in overall beetle abundance or beetle species richness between treatments, but species richness positively covaried with beetle abundance (Table 7). Presence of *N. irrorata* resulted in distinct community composition for the entire beetle assemblage (*p* = 0.047) and the six most abundant species (*p* = 0.001) at the colonization stage (Table 8, Figure 4a,c). Simulating actual predation, the assemblages became even more distinct (Figure 4b,d; *p* = 0.001 for both). There were no differences in beta diversity in any analyses (Table 9). There were no differences in water chemistry across any measured variables (Table 10).

**TABLE 6:** Abundances of beetles in the colonization experiment

Taxa	Abundance	Taxa	Abundance
**Dytiscidae**		**Hydrophilidae**	
*Celina hubbelli*	1	*Berosus exiguus*	1
*Copelatus chevrolati*	3	*Berosus infuscatus*	60
*Copelatus glyphicus*	255	*Cymbiodyta bifidus*	1
*Desmopachria*	1	*Cymbiodyta chamberlaini*	4
*Hydaticus bimarginatus*	2	*Cymbiodyta vindicata*	2
*Hydroporus rufilabris*	2	*Enochrus fimbriatus*	2
*Laccophilus fasciatus*	62	*Enochrus hamiltoni*	7
*Laccophilus proximus*	17	*Enochrus ochraceus*	63
*Neoporus blanchardi*	1	*Enochrus pygmaeus*	8
*Uvarus lacustris*	17	*Helochares maculicollis*	8
**Haliplidae**		*Hydrochara soror*	2
*Peltodytes sexmaculatus*	9	*Paracymus*	720
**Hydraenidae**		*Tropisternus collaris*	9
*Hydraena marginicollis*	3	*Tropisternus lateralis*	46

**TABLE 7:** Results of mixed effects analyses in the colonization experiment for planned contrasts between mesocosms with *N. irrorata* present versus absent and those containing 2 versus 10 *N. irrorata*. Bold indicates statistical significance (*p* < 0.05)

Species	Presence versus absence				2 versus 10			
	Estimate	*SE*	*t*	*p*	Estimate	*SE*	*t*	*p*
*Berosus infuscatus*	0.7533	0.2002	3.763	**0.0010**	0.1894	0.2312	0.819	0.4206
*Copelatus glyphicus*	−1.0740	0.3186	−3.372	**0.0025**	−0.1237	0.3678	−0.336	0.7396
*Enochrus ochraceus*	0.4157	0.2047	2.030	0.0535	0.1155	0.2364	0.489	0.6296
*Laccophilus fasciatus*	−0.5586	0.1606	−3.479	**0.0017**	−0.0923	0.1854	−0.498	0.6626
*Paracymus*	1.2425	0.3902	3.184	**0.0040**	0.6021	0.4505	1.336	0.1940
*Tropisternus lateralis*	0.0514	0.1677	0.307	0.7617	0.0693	0.1936	0.358	0.7236
All beetles	0.6654	0.4494	1.480	0.1518	0.3247	0.5190	0.626	0.5374
Species richness	−0.0731	0.1156	−0.632	0.5327	0.0534	0.1313	0.406	0.6877

**TABLE 8:** PERMANOVA (multivariate centroid location; community composition) results of the four analyses of beetle assemblages in the colonization experiment. Bold indicates statistical significance (*p* < 0.05)

	*df*	SS	Pseudo‐*F*	*p* (perm)	Unique perms
All beetles at colonization					
Treatment	2	918.43	1.7623	**0.047**	999
Block	2	3,437.1	6.595	**0.001**	997
Residuals	22	11,466			
All beetles after predation					
Treatment	2	3,757.2	3.0795	**0.001**	998
Block	2	6,312.1	5.1737	**0.001**	999
Residuals	22	23,490			
6 most common species at colonization					
Treatment	2	1,616.7	3.7236	**0.001**	998
Block	2	5,128.9	11.813	**0.001**	999
Residuals	22	4,776			
6 most common species after predation					
Treatment	2	3,470.6	6.8345	**0.001**	999
Block	2	4,972.1	9.7913	**0.001**	998
Residuals	22	14,029			

**TABLE 9:** PERMDISP (multivariate dispersion; beta diversity) analysis results for the four beetle assemblage analyses in the colonization experiment

Analysis	*df*	*F*	*p*	Permutations
All beetles at colonization	2,24	1.3768	0.342	999
All beetles after predation	2,24	0.6812	0.577	999
6 most common species at colonization	2,24	1.9091	0.213	999
6 most common species after predation	2,24	0.7428	0.583	999

**TABLE 10:** Results of water chemistry analyses from the colonization experiment. The table displays the *t* and *p* values for each analysis across the two contrasts and for when temperature was included as a covariate in the dissolved oxygen analysis

Variable	Presence versus absence		2 versus 10		Temperature	
	*t*	*p*	*t*	*p*	*t*	*p*
Conductivity	0.819	0.4207	1.346	0.1910		
Dissolved oxygen	1.102	0.280	0.751	0.459	−0.693	0.494
pH	1.087	0.287	−0.112	0.911		
Temperature	0.367	0.717	−0.415	0.682		

## 4 DISCUSSION

The consumptive and non‐consumptive effects of predators can vary substantially as a result of variation in both predator and prey traits and their interactions. Prey responses can be morphological, physiological, and/or behavioral, including habitat selection, and we expect these responses to minimize predation risk to prey individuals themselves and/or their offspring. How predators individually affect the myriad of potential prey species in complex communities remains poorly documented in many systems (Hammill et al., 2015). Many predators are relative generalists, preying on anything they can capture, as are *Notonecta*, capturing anything within their occupied microhabitats (e.g., excluding benthic organisms; Streams, 1987; Wilbur, 1997). In lentic freshwater communities, potential prey are often abundant, speciose, and have little (and often no) ability to disperse in response to predators. This includes adult aquatic beetles, which can lose their ability to fly after initial colonization (Johnson, 1969; Zera & Denno, 1997). We expected high mortality rates among taxa that are, at first glance, highly vulnerable to predation. However, among our beetle species, mortality from *Notonecta* ranged from 0% to 95%. These observed species‐specific vulnerabilities were largely a function of morphological and performance traits of each species, relative to those of the predator. Yet, while some species responses at the colonization stage matched their vulnerability, others did not, including a highly vulnerable species that actively preferred predator patches.

**FIGURE 5:**
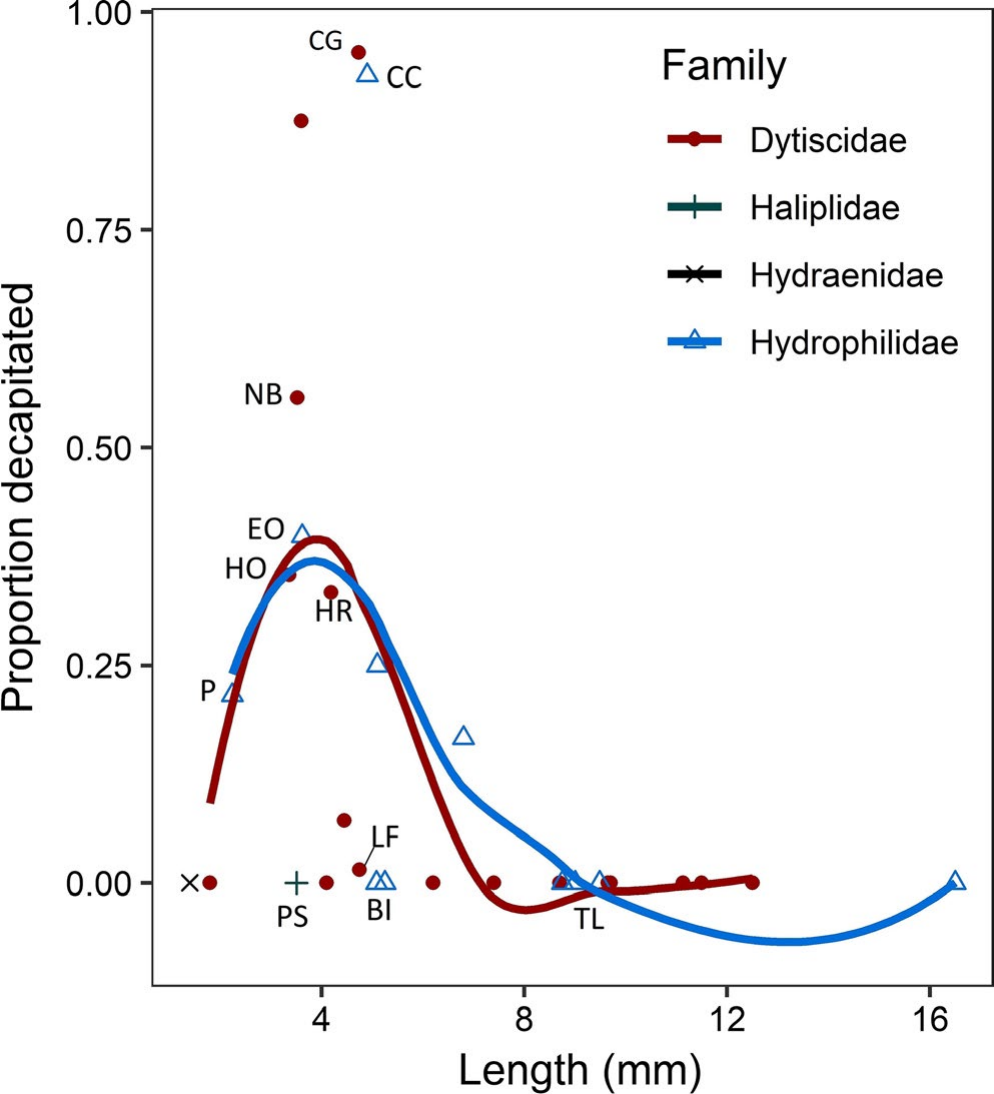
Scatterplot of decapitation rates by length for all species with abundances > 3 in the beetle predation study (Table 3). Mean size for common species (labeled; see Figure 1 for abbreviations) is from our beetle trait results; approximate mean sizes for other species are based on published figures (Epler, 2010). Decapitation rate increases with size to intermediate sized species (BI, CC), when excluding species not vulnerable to decapitation due to faster swimming speeds (LF, BI) or higher force requirements for decapitation (PS). The two lines for Dytiscidae and Hydrophilidae are fit with geom_smooth(method = “loess”) in ggplot2 for illustrative purposes only, while only single species of Haliplidae and Hydraenidae were present. This figure is intended to be illustrative of overall patterns that could be formally tested if all species had higher abundances, and thus greater confidence in decapitation rates

Trait differences among beetle species clearly related to vulnerabilities: species that were larger, faster, and had more resistant morphology had lower vulnerability to *N. irrorata*. *Peltodytes sexmaculatus* had by far the highest force required for decapitation, suggesting this trait alone was sufficient protection. Oddly, seemingly vulnerable smaller species, particularly *Paracymus* and *E. ochraceus*, that were slow swimmers and not physically resistant to predation, also had reduced predation rates. For predators with considerable prey handling times like *Notonecta* (Streams, 1987), optimal foraging should operate primarily across a gradient of prey size rather than prey density (Charnov, 1976; Werner & Hall, 1974). Thus, predation by *N. irrorata* varies across the size gradient for beetles (Figure 5), with moderate‐sized taxa experiencing the highest mortality rates, though other traits can override this.

Regardless of their vulnerability, beetles can avoid potential predators through habitat selection at the colonization stage. Habitat selection should maximize expected fitness by matching species’ habitat preferences with the risk posed by those predators to adults and/or their offspring (Rieger et al., 2004; Thompson, 1988), yet we observed a wide range of colonization responses to *N. irrorata*. Only three species (of six) reduced colonization in the presence of *N. irrorata*: *Paracymus* and *E*. *ochraceus* were moderately vulnerable to predation as adults, so avoidance would be expected, but avoidance by seemingly invulnerable adult *B. infuscatus* is surprising. The explanation may lie in the fact that beetles select habitats for both themselves and their offspring. Larval beetles have soft exoskeletons, and are generally very active, but are slower swimmers, theoretically placing them at greater risk of predation than adults. In fact, given positive size‐dependent prey selection in *N. irrorata* (Figure 5), larval vulnerability may be positively correlated with size across the species represented here. Thus, the lack of response by *T. lateralis* matches adult, but likely does not match offspring, vulnerability; *T. lateralis* have shown a similar lack of colonization response to a small predatory fish (Resetarits & Pintar, 2016). Colonization by adult beetles generally determines oviposition site choice, as secondary dispersal is costly and often impossible, especially for females, though actual oviposition behavior is difficult to study in many aquatic beetles (Binckley & Resetarits, 2007; Resetarits, 2001). This illustrates a difficult, unanswered question, which is whether ovipositing, fully aquatic species select habitats for themselves, their offspring, or both, and how those considerations are prioritized.

Most perplexing results are the two species, both dytiscids, that colonized mesocosms containing *N. irrorata* at higher rates: *L. fasciatus* was largely unaffected by predation, but *C. glyphicus* had >95% mortality. These two species respond to a variety of patch characteristics (other predators, leaf litter, potential prey, water quality, etc.; Pintar et al., 2018; Pintar & Resetarits, 2017a, 2017b; Resetarits & Pintar, 2016; Resetarits et al., 2019), but none of the prior work explains the patterns observed here. We then considered the possibility that *N. irrorata* produces an attractant (chemical mimicry; Akino et al., 1999; Johnstone, 1996). Prior work on other species in these genera identified two insect semiochemicals, 4‐hydroxybenzaldehyde and me‐4‐hydroxybenzoate, found in *Notonecta*, *Copelatus*, and *Laccophilus*, but not other dytiscid or hemipteran taxa, or any hydrophilids (Dettner, 1979, 1985; El‐Sayed, 2019; Pattenden & Staddon, 1968; Schildknecht, 1970; Staddon, 1979). These semiochemicals are thought to be *Notonecta* pheromones, both allomones and pheromones in *Copelatus*, and *Laccophilus* allomones, though roles are not completely understood for all species. Thus, although we did not directly test this hypothesis or verify the presence of these semiochemicals here, the same semiochemicals may serve similar purposes in the three taxa (mate attraction, defense), but may also serve as conspecific attractants, with potentially severe consequences for *Copelatus*. Other potential reasons for these counterintuitive patterns, such as presence of conspecifics, heterospecifics, or prey are not supported by our results or prior studies. Potential tradeoffs between adult and larval survival are also unlikely as larvae should be even more vulnerable to *Notonecta* than adults.

Habitat selection models typically assume accurate assessment of available patches and optimal selection of the best available patch (Fretwell & Lucas, 1969). If the cues produced by *N. irrorata* are unreliable, or misleading, their presence may constitute an ecological trap (Delibes et al., 2001; Schlaepfer et al., 2002): cues originally known to act as pheromones and produced by the predator are potentially attracting prey, also acting as allomones. Olfactory cues are ubiquitous in insect communication, and predators often exploit prey pheromones for detection and location of prey (Svensson et al., 2004; Zuk & Kolluru, 1998), but potential mimicry of prey pheromones, or general attraction of prey by predators, is not well documented (Haynes et al., 2002; Haynes & Yeargan, 1999), especially in aquatic systems. Given the myriad compounds occurring in insect semiochemicals, it is possible that these three taxa simply converged on a similar communication cue. In any case, if cues are poor predictors of patch quality, the result is non‐ideal habitat selection (Arlt & Pärt, 2007). Choosing a habitat with increased mortality should not be adaptive under any scenario, and may simply be a case of maladaptation, which may be more common than previously recognized (Brady et al., 2019).

As three of the most abundant aquatic insect species at UMFS, *N. irrorata*, *C. glyphicus*, and *L. fasciatus* are often found in temporary ponds (but especially *C. glyphicus*; (Miller & Bergsten, 2016) and coexist at the landscape scale despite the seemingly maladaptive habitat selection patterns. Due to this co‐occurrence, it could theoretically be possible that *N. irrorata* serve as a proxy for fishless habitats, attracting *C. glyphicus* and *L. fasciatus*. However, this seems highly unlikely as all three taxa have direct, strong responses to the presence/absence of fish, as do most of the other beetle species in our habitat selection experiment (Resetarits et al., 2019), including those that had opposite responses to *N. irrorata* (*E. ochraceus, B. infuscatus*, *Paracymus*). Other patch characteristics may be more important in complex situations, although predation risk has often, but not always, shown primacy over other factors (Binckley & Resetarits, 2008; Pintar et al., 2018). Presence of conspecifics or heterospecifics could play roles in community contexts, but colonizing adult beetles largely do not respond to the presence of other beetles within patches (Pintar & Resetarits, 2020a). Interestingly, when a range of habitat patch sizes is available, *N. irrorata* selects the largest available patches and *C. glyphicus* the smallest, whereas *L. fasciatus* shows no preferences (Resetarits et al., 2019). Thus, spatial sorting in which patch size acts a niche dimension (and often relates to hydroperiod) may mediate the attractiveness of *N. irrorata*, particularly for *C. glyphicus*, especially as patch size may be determined at greater distances than chemical cues.

*Notonecta* and most aquatic beetles strongly avoid habitats containing fish, greatly reducing the landscape‐level availability of habitats for colonization (Resetarits & Pintar, 2016; Resetarits et al., 2019). In fishless habitats at UMFS, *Notonecta* (mostly *N. irrorata* and *N. indica*) are common and present in many, if not most, habitat patches, particularly in summer. *Notonecta* are perhaps the most important predators of adult aquatic beetles in fishless habitats, while other taxa, with the exception of certain larval salamanders, have little to no effect. Colonization decisions by beetles can be critical determinants of fitness, as secondary dispersal for oviposition (days or weeks later) may be unlikely as flight is costly and many taxa autolyze wing muscles for reproduction (Johnson, 1969; Zera & Denno, 1997). Thus, making poor colonization decisions likely reduces expected fitness.

The role of predators in structuring communities is widely recognized, but traditionally focused on post‐colonization processes. While non‐consumptive effects of predators have had increased attention in recent decades (Relyea, 2001; Resetarits & Wilbur, 1989; Werner et al., 1983), the relative contributions of non‐consumptive and consumptive effects to observed patterns of species abundances and distributions, in particular, is poorly understood in many systems, especially those with diverse groups of interacting species (Creel et al., 2017; Vonesh et al., 2009). Clearly, the relative importance to population growth of direct predation versus non‐consumptive responses to predation risk can vary among species (LaManna & Martin, 2017). Measurements of predation sensu *stricto* alone have often limited our understanding of predation risk (Lank & Ydenberg, 2003), as has the fact that predator‐avoidance responses are often not measured in equivalent ways to predation (Creel et al., 2017). We addressed these issues by determining individual‐level predation rates and habitat selection decisions, creating a complete picture of both the initial non‐consumptive habitat selection effects and final predation rates of *N. irrorata* in a diverse group of aquatic insects. It is possible, and perhaps likely, that our taxa have additional predator‐avoidance responses within the aquatic stages, such as changes in behavior, microhabitat use, or oviposition decisions that were not measured. All of these additional responses are preceded by habitat selection and contribute to the observed mortality rates; clearly any post‐colonization predator‐avoidance responses by adults, if they exist, are not effective in the most vulnerable taxa (*C. glyphicus*, *C. chamberlaini*). While we have built on existing evidence that both consumptive and non‐consumptive effects can vary tremendously in their relative importance, we have added relatively novel evidence that, at least under the scenarios we presented, non‐consumptive effects at the habitat selection stage can generate natural ecological traps.

Overall, we observed variation in consumptive effects of a predator on prey species, variation in prey antipredator traits, and a variety of functionally unique responses to an important predator at the colonization stage. Relationships between predation risk and habitat selection decisions varied among species, with species‐specific traits, vulnerabilities, and responses to predator chemical cues contributing to variation in consumptive and non‐consumptive effects. The diversity of observed responses, and the strong signal of non‐consumptive effects seen here, challenges us to re‐imagine our approach to understanding how predators impact populations, communities, and metacommunities. Clearly, if we only considered consumptive effects of predators, or conversely only non‐consumptive effects, we would not have complete or accurate perspectives on how predators contribute to the distributions of species. It emphasizes the need to determine species‐specific effects for both predation risk and predator‐avoidance responses in order completely understand predator–prey interactions and processes generating observed patterns of abundance and community structure.

## CONFLICT OF INTEREST

The authors declare no conflicts of interest.

## AUTHOR CONTRIBUTIONS

**Matthew R. Pintar:** Conceptualization (lead); Data curation (lead); Formal analysis (lead); Investigation (lead); Methodology (lead); Visualization (lead); Writing‐original draft (lead); Writing‐review & editing (lead). **William J. Resetarits:** Formal analysis (supporting); Funding acquisition (lead); Investigation (supporting); Methodology (supporting); Resources (lead); Supervision (lead); Visualization (supporting); Writing‐review & editing (supporting).

### OPEN RESEARCH BADGES

This article has earned an Open Data Badge for making publicly available the digitally‐shareable data necessary to reproduce the reported results. The data is available at https://doi.org/10.5061/dryad.xksn02vf6.

## Data Availability

Data are available in Dryad at https://doi.org/10.5061/dryad.xksn02vf6.
